# Estimating mortality rates among passerines caught for ringing with mist nets using data from previously ringed birds

**DOI:** 10.1002/ece3.4032

**Published:** 2018-04-26

**Authors:** Gary D. Clewley, Robert A. Robinson, Jacquie A. Clark

**Affiliations:** ^1^ BTO The Nunnery Thetford UK

**Keywords:** avian, bird ringing, injury, mark–recapture, mortality, passerine, trapping, welfare

## Abstract

Mist netting is the most commonly used method for catching birds for scientific ringing, but despite decades of use, there have been few attempts to quantify the associated potential risks to the individuals caught. Any incidence of mortality through capture and handling, however low, is of potential ethical concern and may also introduce biases into the data. We estimate the mortality rate associated with capture of previously ringed (recaptured) passerines from the British and Irish Ringing Scheme (c. 1.5 million records) caught using mist nets. The importance of species, age, mass, month, time, previous captures, and an index of predator occurrence was tested using generalized linear mixed‐effects models. The average mortality rate was 0.0011, most of which was reported to occur before the individuals had been extracted from the nets (c. 70% of incidents). Juveniles appeared to be at higher risk and the incidence of predation from mist nets was seasonal, with increased risk during the winter. Species differed in their reported mortality rates with the apparent risk being greatest for Chiffchaff *Phylloscopus collybita* (0.0029) and Bullfinch *Pyrrhula pyrrhula* (0.0027). To improve our understanding (and hence minimize risk in future), we recommend collecting more complete data on incidences of mortality, and also injuries; exercising increased care when the species we have identified as being at greater risk are likely to be captured, and ensuring there are robust procedures for the checking of nets (as most reported incidents of mortality occur before handling). We also recommend that all Ringing Schemes should collate and make available data on capture‐related mortality. Overall rates of mortality associated with capture, although, were low and support the use of mist netting as a safe capture technique, without undue bias from mortality, when used by appropriately trained individuals.

## 1 INTRODUCTION

Individually marking and reencountering wild animals is a key component of many areas of biological research. It is essential, however, to consider whether there may be any impact of such activities both on individuals themselves and on the populations of which they are a part. Wild birds have been marked with metal rings (bands) as part of scientific ringing programs throughout the world for over a century to quantify demographic processes and movements at a range of scales (e.g., Baillie & Schaub, 2009; Clark, Thorup, & Stroud, 2009; Ralph & Dunn, 2004). Furthermore, ringing data have informed a wide variety of other ecological and conservation research programmes (Anderson & Green, 2009), for example, diet, seed dispersal and genetics (González‐Varo, Arroyo, & Jordano, 2014), phenology (Reed, Jenouvrier, & Visser, 2013), moult (Newton, 2009), or host–parasite relationships (Møller et al., 2013). Any effect of capturing birds is not only an important ethical consideration (Wilson & McMahon, 2006), as the welfare of those handled must be a priority, but also may influence the integrity of the data collected, as biases may be introduced if capture and handling cause changes in behavior or survival, especially where these differ nonrandomly among groups (e.g., in age or sex). Ultimately, the benefit of the information gained when capturing wild animals for study needs to outweigh the potential risk to the individuals which are caught (Anon 2012).

Potentially adverse effects that may cause injury or mortality could occur either as a direct result of the capture and handling event, or subsequently over a longer period through a cost of bearing a ring or device, for example, by increased energetic demands (Wilson & McMahon, 2006) or direct injury (Pierce, Stevens, Mulder, & Salewski, 2007). The effect of the addition of marks and devices to birds has been assessed previously (Calvo & Furness, 1992), particularly for color marks (Splittgerber & Clarke, 2006) and data‐logging or tracking devices (e.g., Barron, Brawn, & Weatherhead, 2010; Bowlin et al., 2010; Constantini & Møller, 2013; Thaxter et al., 2016), as has the physiological response to capture (e.g., Romero & Romero, 2002). However, the direct effects of the capture methods themselves have received less attention and few quantitative estimates of injury or mortality have been published.

**Figure 1:**
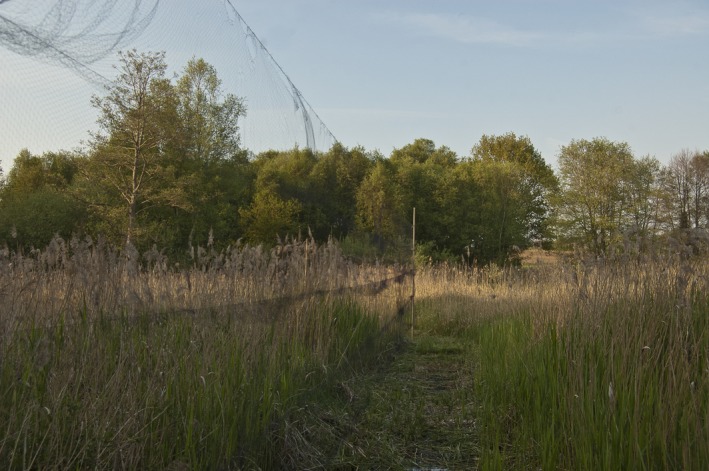
Mist nets are widely used to catch passerine birds in scrub and other habitats

Mist netting is widely used as a method for the capture of birds (Figure 1). In recent years, for example, around a million birds annually have been captured for ringing in Britain and Ireland; most of these (85%–90%) are passerines caught in mist nets and, additionally, there are usually over 200,000 recaptures of ringed individuals (Robinson, Leech, & Clark, 2017). Mist netting is considered among researchers and bird‐ringing organizations to be safe and effective when carried out by trained and experienced individuals who follow published guidelines (e.g., Busse & Meissner, 2015; Gustafson, Hildenbrand, & Metras, 1997; Redfern & Clark, 2001). Spotswood et al. (2012) carried out an assessment of mortalities associated with mist netting using data from five banding organizations across North America, while other examples of reported mortality rates for mist netting are derived from very few captures or locations (Brooks, 2000; Duarte, 2013; Recher, Gowing, & Armstrong, 1985; Stamm, Davis, & Robbins, 1960). Defining any threshold of “acceptable” mortality in the research of wild birds is a difficult and sensitive issue and few organisations have attempted to do so, although Ralph, Geupel, Pyle, Martin, and DeSante (1993) recommended that ringing practices should be scrutinized if mortality exceeds an average of 1%.

**Figure 2:**
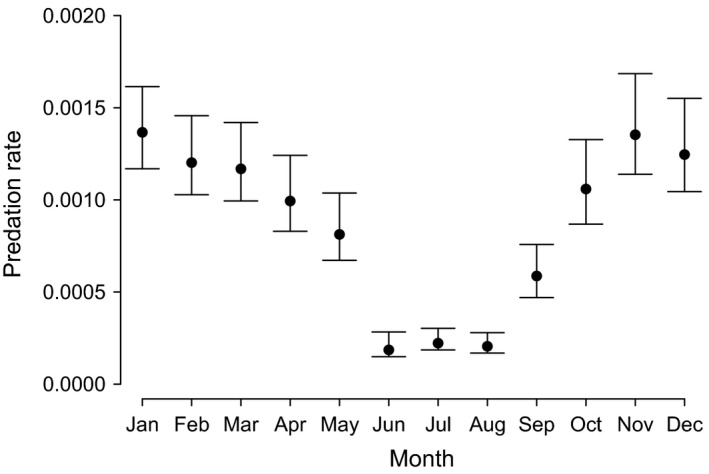
Estimated average rate of individuals reported to have been killed by predators when in mist nets in each month, back‐transformed (inverse logit) from top selected logistic regression model; bars indicate ±1*SE*

This study quantifies the reported mortality rate among common passerine species recaptured using mist nets, using data submitted to the British Trust for Ornithology (BTO) Ringing Scheme. Factors which may influence the likelihood of mortality are investigated including intrinsic factors such as the age of an individual, as well as those of the capture environment such as time of day/year and the presence of predators. By reporting this information, we aim to encourage wider evaluation of the incidence of mortality associated with ringing activities in order to inform guidance to help minimize the risks.

## 2 MATERIALS AND METHODS

### 2.1 Data

Quantifying mortality poses some difficulties, as we rely on ringers (banders) self‐reporting incidents. Nevertheless, in Britain and Ireland, all ringers are asked to report all dead ringed birds and provide information on the circumstances, regardless of how they died. For this analysis, we estimate the risk of mortality during the data collection process for birds that were recaptured, having been ringed on a previous occasion. This was necessary because, within the BTO Ringing Scheme, there has not been a mechanism for recording data on mortality of birds at the time of first capture, that is, without an associated ring number, due to the structure of its database.

We included data from 2005 to 2013, as data from all recaptured individuals were available electronically, with 1,565,743 recapture records of 166 passerine species from across Britain and Ireland. Over the 9‐year period, some individuals were recaptured multiple times (57% one recapture, 20% two recaptures, and 23% three or more recaptures). The number of mortality incidents (*n* = 1,646) was determined by selecting records coded as EURING Circumstance code 08 (“Ringing casualty,” EURING 2015). We used a free‐text field associated with each recapture to categorize the apparent cause of death and, where possible, the stage of the capture process at which the mortality occurred. In about half of all records of mortality, any associated information was either ambiguous or missing and these records were categorized as “unknown.” Information on nonfatal injuries has not been systematically collected, so this analysis considers mortality events only.

### 2.2 Data analysis

All analyses were carried out in R 3.0.2 (R Core Team 2013). A binary response variable (Mortality/No mortality) for each record was modeled using generalized linear mixed‐effects models (GLMM) with Laplace approximation, specifying a binomial error distribution and logit link function with the *lme4* package (Bates, Maechler, Bolker, & Walker, 2014). Being predated while in the mist net (including attack by species not typically considered predators of birds) was identified as the cause of mortality in 44% of cases, these records were analyzed separately from all other records as the predictors of mortality risk may differ. The majority of other records (totaling 50%) had no known cause of death and were pooled with records where causes other than predation were confirmed (c. 6% of records). The effect of possible predictor variables on the probability of mortality (species, age, mass, time of day, month, and number of previous captures) was investigated as fixed effects. Sparrowhawks *Accipiter nisus* are among the commonest predators of a wide range of passerine species (Newton, 1986); therefore, relative Sparrowhawk occurrence was also included (as a fixed effect) in models for the predated response.

Only individuals that could be assigned one of the following age classes at each capture event (based on reported age at time of recapture or first encounter) were included in the analyses; “juvenile” (EURING Age 3 and Plumage code J), “first‐year,” that is, in their first calendar year and completed their postjuvenile moult (EURING Age 3), or “adult” (EURING Age 4, 5 or 6; EURING 2015). Mass was estimated as the average values for individual species from Robinson (2005) as few individuals were weighed postmortem. Relative Sparrowhawk occurrence was defined as the proportion of tetrads in a 10 km square surveyed during the BTO Bird Atlas 2007‐2011 (Balmer et al., 2013) in which Sparrowhawk was recorded. Time of day (hour) and number of previous captures were included as continuous variables, as was month as a categorical factor.

Approximately 5% of the dataset had missing values for the time of day or relative Sparrowhawk occurrence variables, which would prevent multiple models being compared using the exact same dataset (Nakagawa & Freckleton, 2011). Missing values for these variables were therefore imputed with the mean. Sex was not known for 46% of all records and therefore was not included in the main analyses, although a supplementary analysis using nine species which have sexually dimorphic plumage, and so the sex of most individuals was recorded, was carried out (Appendix S1). To identify the species most at risk, only species with ≥20 reported mortalities were included in the analyses (predated response; *n* = 601, “other” response; *n* = 826, no mortalities; *n* = 1,258,438).

We selected a candidate set of models a priori (19 for the predated subset and 15 for “other”) which included various combinations of the seven fixed effects (Appendix S2). All models included year, location (10 km square of the Ordnance Survey National Grid), to account for spatial and temporal clustering in the dataset, and bird family, to account for phylogeny, as categorical random effects. For each response, all models were compared on the basis of their Akaike information criterion accounting for sample size (AICc) and Akaike weight (*w*_*i*_) following guidelines in Bolker et al. (2009). Parameter estimates were determined by averaging all models with ΔAICc < 7 from the best fit model using the *MuMIn* package (Barton, 2014), as all models in this set may be informative (Burnham, Anderson, & Huyvaert, 2011).

## 3 RESULTS

The mortality rate associated with capture varied among species (Table 1) with 40% exhibiting at least one incidence and an overall rate of 0.0011 (annual means varied between 0.0009 and 0.0012). The single largest identified cause of death was predation, mostly by raptors (Table 2). The majority of mortalities, regardless of cause, were reported to occur before the individual was removed from the mist net. No capture‐related mortality was recorded for 70 species (which had a mean of 228 and a maximum of 4,866 live recaptures per species).

**Table 1:** Total ringing mortality rates across 9 years (2005–2013) for passerines recaptured using mist nets in Britain and Ireland ordered by reported mortality rate. Species with fewer than 20 reported mortalities (*n* = 26) and species recaptured with no reported mortalities (*n* = 70) were aggregated as “Other species”. “Other” includes mortalities of unknown cause

Species	Mortalities			Total recaptures	Mortality rate
	Predated	“Other”	Total		
Chiffchaff *Phylloscopus collybita*	18	58	76	26,614	0.0029
Bullfinch *Pyrrhula pyrrhula*	16	45	61	22,461	0.0027
Lesser Redpoll *Acanthis cabaret*	9	19	28	11,956	0.0023
Willow Warbler *Pylloscopus trochilus*	8	30	38	19,899	0.0019
Goldcrest *Regulus regulus*	5	26	31	16,972	0.0018
Wren *Troglodytes troglodytes*	21	47	68	43,319	0.0016
Coal Tit *Periparus ater*	20	75	95	62,596	0.0015
Greenfinch *Chloris chloris*	32	27	59	38,567	0.0015
Blue Tit *Cyanistes caeruleus*	139	248	387	311,068	0.0012
Chaffinch *Fringilla coelebs*	25	35	60	56,728	0.0011
House Sparrow *Passer domesticus*	17	5	22	19,416	0.0011
Robin *Erithacus rubecula*	48	29	77	84,250	0.0009
Dunnock *Prunella modularis*	50	26	76	91,400	0.0008
Blackbird *Turdus merula*	42	23	65	80,502	0.0008
Blackcap *Sylvia atricapilla*	14	12	26	32,364	0.0008
Great Tit *Parus major*	111	55	166	232,757	0.0007
Long‐tailed Tit *Aegithalos caudatus*	22	28	50	69,946	0.0007
Reed Warbler *Acrocephalus scirpaceus*	10	27	37	64,848	0.0006
Siskin *Spinus spinus*	16	14	30	46,648	0.0006
Goldfinch *Carduelis carduelis*	9	13	22	35,393	0.0006
Other species	85	87	172	198,039	0.0009
Total	717	929	1,646	1,565,743	0.0011

**Table 2:** Reported causes of ringing mortality for different stages of the data collection process. “Net” refers to individuals caught in mist nets; “Holding” is when individuals were placed individually in fabric bags; and “Processing” includes reading the ring and taking measurements. A ‘—’ indicates the cause of death is not considered relevant for the data collection stage. Records were only categorized if the additional information provided by ringers was unambiguous

Cause of death	Data collection stage						Total
	Net	Extraction	Holding	Processing	Release	Unknown	
Predation—Raptor/Owl	425	—	0	—	0	0	425
Predation—Other bird	82	—	0	—	0	0	82
Predation—Mammal	110	—	0	—	0	0	110
Predation—Other	6	—	0	—	0	0	6
Predation—Unknown	94	—	0	—	0	0	94
Tangled	63	0	—	—	—	0	63
Cold	5	0	0	0	0	2	7
Internal injury	1	0	0	0	0	4	5
Poor condition	9	1	2	1	0	3	16
Handling accident	0	0	0	0	0	1	1
Other	3	0	0	0	1	0	4
Unknown	336	5	88	66	17	321	833
Total	1,134	6	90	67	18	331	1,646

Similar models were selected for both the confirmed predated and “other” causes datasets: The risk of attack or predation in the net varied by age and time of year (ΔAICc of next model = 108.1) and other mortality varied by age and species (ΔAICc = 185.7, Appendix S2). There was a sharp decline in the rate of predation during the summer months, increasing again to a peak in winter (Figure 1). Juveniles were at relatively higher risk of predation (β ± *SE* = 2.46 ± 0.15) than adults; however, there was no significant difference between adults and first‐years (β = 0.09 ± 0.13). The most commonly reported predator was Sparrowhawk (40% of incidents), but a range of other avian (23%) and mammalian (15%) species were also reported (Appendix S3).

Using the dataset where birds were not confirmed to have been predated, the probability of an individual dying varied with age with both juveniles and first‐years having a higher likelihood of mortality than adults (juveniles: β = 1.43 ± 0.09; first‐years: β = 0.32 ± 0.10), an increase in the mean mortality rate from 0.0003 for adults to 0.0011 and 0.0004, respectively. Species differences were also detected (Table 1), but there was no evidence of a seasonal pattern (Appendix S2). Analyzing the data only using a subset of species which were sexually dimorphic did not affect overall interpretation of the results (Appendix S1).

## 4 DISCUSSION

We found the overall reported mortality rate for passerines recaptured in mist nets in recent years to be 0.0011, although the likelihood of mortality varied according to species, age, and season. Our estimate for ringing mortalities in Britain and Ireland is lower than that estimated in North America (0.0023 ± 0.0015, Spotswood et al., 2012). In both studies, the cause and stage of death was not reported in many cases, hampering our ability to determine the relative importance of intrinsic (due to the individual's condition) and extrinsic (such as differences in training practices or type of mist net) factors in these incidents; more systematic reporting of incidents should be encouraged in the future. In many cases, although, the exact cause of death will be unknown, as it is generally impracticable to undertake routine postmortem examinations. Spotswood et al. (2012) reported “stress” as the largest contributor to mortality. Although stress is difficult to define, and almost certainly results from a variety of underlying conditions, it might account for mortalities with no other obvious cause (which also formed a large proportion of incidents we considered).

In North America, larger passerine and near‐passerine species were more prone to predation when in mist nets, by a range of common predators (Spotswood et al., 2012), and it was concluded that this was related to their conspicuousness during capture, either by making more noise or simply being more visible. Neither species nor mass was found to be related to the risk of predation in Britain and Ireland, although the majority of mortalities reported occurred while birds were still in mist nets and predation is a considerable component of the overall risk, which we also found to vary seasonally. The increased level of predation in winter may reflect a shift in habitat use, or a greater willingness for predators to hunt in proximity to the human activity associated with mist netting. Alternatively, changes in ringing practice could explain different predation rates if, for example, there are changes in preferred habitats where ringers mist net or increased provisioning of food. It may be expected that concentrations of passerines using bird feeders in winter could result in increased predation risk. However, Roth and Lima (2007) demonstrated that Sharp‐shinned Hawk *Accipiter striatus* in North America did not hunt repeatedly and predictably in particular areas of high passerine abundance, which would reduce the likelihood of prey species developing avoidance mechanisms. This suggests it may be difficult to predict in advance when or where predation events from mist nets are more likely to occur, even if ringing activities are concentrating individuals with various types of lure.

For cases where predation was not reported to be the cause of mortality, species differences were important and are reflected in the mortality rate variation observed (Table 1). Overall, average species mass was not an important predictor of risk, but several of the smallest species captured, including Chiffchaff, Willow Warbler *Phylloscopus trochilus*, Goldcrest *Regulus regulus* and Wren *Troglodytes troglodytes,* did show higher than average mortality rates. Other species appear to exhibit particular susceptibilities, for example, Bullfinch had an elevated mortality rate, with reports of apparent lung hemorrhage during handling (Redfern & Clark, 2001), although why this should be the case is apparently unknown.

Age was an important predictor of mortality with juvenile (recently fledged) birds apparently the most vulnerable. Estimates of postfledging mortality can be high for some passerine species, for example, natural daily mortality rates of Great Tit *Parus major* of c. 0.10 in the first few days after fledging, reducing to 0.01–0.03 in subsequent weeks (Naef‐Daenzer, Widmer, & Nuber, 2001). Until they have completed their postjuvenile moult, the plumage of young birds is more loosely textured and there are fewer body contour feathers compared with adults (Ginn & Melville, 1983), which will affect their ability to remain insulated, especially when resting still in a net (Newton, 1968), and may make them more susceptible.

Spotswood et al. (2012) reported that individuals which were captured more often had lower incidence of mortality and suggested they were likely to be fitter compared with transient individuals, the latter both not holding a territory and, by definition, having a lower probability of recapture. However, other studies show that birds appear to learn to avoid nets once caught, at least for a short while (Simons, Winney, Nakagawa, Burke, & Schroeder, 2015), and that individuals in poorer condition may be captured more frequently, as they are less able to avoid capture (Al Rubiaee, Al‐Murayati, & Møller, 2017). We did not find any relationship between the number of previous captures and the risk of mortality, although all the birds in our study had to have been captured once before entering the dataset. For historical reasons, we were unable to capture information on the mortality of unringed individuals; however, considering only recaptures has still been useful. Recaptures provide information on approximate mortality rates, relative differences between species, and a benchmark against which future data can be judged. Other mark–recapture schemes could also benefit from investigating and publishing findings from similar data even if information from unmarked individuals is missing.

While it is important to understand, and minimize, the scale of any effects, it is unlikely that a zero mortality rate will ever be possible when trapping and handing wild animals for scientific purposes. For example, in our study, Blue Tit *Cyanistes caeruleus* was the most commonly recaptured species and had the highest number of reported mortalities with a mean of 48 per year (of all ages). Given the British population of Blue Tit is estimated to be 3.4 million breeding pairs (Musgrove et al., 2013), the estimated mean natural adult daily mortality rate (0.0017, Siriwardena, Baillie, & Wilson, 1998) implies that, on average, approximately 11,500 adult individuals die each day. It is clear that the mortality risk associated with mist netting will be negligible at a population level, even if mortality were completely additive, whereas in reality, it is likely to be at least partly compensatory, that is, the rates of alternative causes of mortality may fall correspondingly (Burnham & Anderson, 1984). This may particularly be the case for individuals who were in poor condition when caught and at least some of these individuals for which no specific cause of death was apparent may have died for reasons unconnected with capture. There is some evidence, for example, that individuals with higher bacterial infection loads are less likely to evade capture (Al Rubiaee et al., 2017). However, a lack of population‐level effects does not negate welfare concerns for individuals and, as we demonstrate here, efforts should be made to identify species or groups most at risk.

In order for bird ringing to continue to be a valid method to monitor and study wild populations, in an ethical way, wider knowledge and discussion among practitioners of the potential costs to captured birds is essential. Wilson and McMahon (2006) discussed the ethical issues surrounding the attachment of measuring devices to wild‐caught animals. They highlight several key ways in which problems of perception may arise, which are equally applicable to the use of mist nets and other capture techniques for bird ringing. For example, there may be a failure on behalf of the researcher or practitioner to communicate the motivation and value of the work being carried out. They also suggest that transparency regarding research activities may ease concerns and allow a more empirical appraisal of practice. Where issues are identified steps should be implemented to mitigate the risks, for example, the BTO scheme (among others) requires that those licensed to attach devices, or use nonstandard marks, monitor and report annually on any observed impacts.

It is important to note that we have only considered incidence of mortality, data have not been collected to date on sublethal injury rates. Incidences of ring‐related injuries have been reported for some species (e.g., Amat, 1999; Griesser et al., 2012) but, importantly, the causes have been identified and advice provided on prevention. These gaps in our knowledge, resulting from an absence of applicable data being collected in the past, could be addressed in the future. Additionally, reporting cases where there is evidence that injuries or negative effects are rare or absent (e.g., Broughton, 2015; Cresswell, Lind, Quinn, Minderman, & Whitfield, 2007) should also be encouraged.

As with mist netting, there are few examples of published evaluations of the safety of other methods for trapping wild birds; exceptions include published mortality rates of wildfowl caught using various methods in North America (0.0017–0.0116, Dieter, Murano, & Galster, 2009) and the United Kingdom (0.0010, O'Brien, Lee, Cromie, & Brown, 2016). However, in the context of the capture and handling of wild animals more generally, mist netting of passerines appears to be a low‐risk activity. By comparison, reported mortality rates for small mammal trapping vary considerably depending on the method used (Drickamer & Paine, 1992; Edwards & Jones, 2014; Karraker, 2001), but an assessment of 68 surveys in Australia reported a rate of 0.017 using box traps for terrestrial mammals and 0.0036 for bats captured in harp traps (Lemckert, Brassil, Kavanagh, & Law, 2006). The mortality rate in studies of large mammals can also exceed 0.03 (Arnemo et al., 2006). Trap performance criteria have been proposed for mammals (Powell & Proulx, 2003); however, there is a general scarcity of information and published rates may not be widely representative. Researchers need to openly assess and report the impacts of their study methods in ways that are amenable to meta‐analysis (Arnqvist & Wooster, 1995). This study provides a way of using existing data to give an informative measure of relative risk of different species and identifies improvements for future practice, which can be used by ringing schemes around the world.

For those involved (either volunteer or professional) in the monitoring and capture of wild animals, there is a responsibility to maintain high standards, including the rigorous and transparent reporting of any capture‐related mortality (Byrne, O'Keeffe, Fogarty, Rooney, & Martin, 2015). The overall mortality rates presented here can only represent minimum estimates, given the unavoidable bias from under reporting and lack of data on injuries. Nevertheless, mortality rates, as derived from the most extensive dataset currently available, are low and are unlikely to be a significant source of bias in analyses.

In light of our findings, we recommend that:

- Smaller species and those with the highest incident rates (e.g., Chiffchaff and Bullfinch) should be prioritised during ringing activities to minimize handling time; as should juveniles of any species where these are readily identifiable.
- Where practical, species identified as higher risk should only be handled by personnel who have gained sufficient experience to do so safely; handling ability and experience have been shown to in influence the incidence of injury and mortality (Moore, 2003; Recher et al., 1985). Appropriate training protocols should be implemented that highlights the situations, such as time of year, where risks are greatest.
- Efficient net checking protocols should be considered to be particularly important to reduce the number of incidents (Busse & Meissner, 2015) given the mortality rates experienced before individuals are extracted from the net, which, in this study at least, are exacerbated by higher predation rates during winter. Redfern and Clark (2001) indicate that visits to mist nets catching passerines should be no more than 30 min apart under ideal weather conditions and more frequent when the catching rate is high, temperatures are extreme, or predators are likely to be active. Catching effort should be adapted to the number and skills of the personnel available, which may necessitate closing of some or all nets if conditions become less than optimal.

More generally, we recommend that any projects working with animals in the wild, including ringing schemes, should encourage the collection of both mortality and injury information (if not already doing so) and report on these data periodically. If ringing schemes have yet to collect full data on injury and mortality, including from unmarked individuals, then comparable analyses to those described in this paper using recapture data to assess overall rates would provide a fuller picture of the risks associated with capture. The results should be fed back to both ringers (to make them aware of higher risk species and situations) and analysts (if any data set might be biased). This will allow any areas of concern to be identified and addressed and specific guidance to be disseminated as appropriate.

## CONFLICT OF INTEREST

None declared.

## AUTHOR CONTRIBUTIONS

JAC conceived the idea. RAR and GDC designed the methodology; GDC analyzed the data and led the writing of the manuscript. All authors contributed critically to the drafts and gave final approval for publication.

## DATA ACCESSIBILITY

Data available from the Dryad Digital Repository: https://doi.org/10.5061/dryad.h0dj3p3.
