# Supplementary material for: Estimating mortality rates among passerines caught for ringing with mist nets using data from previously ringed birds
Source: Ecol Evol. 2018 Apr 26;8(10):5164–72. doi: 10.1002/ece3.4032 (PMC5980556; doi:10.1002/ece3.4032)
Supplement: Supplementary file 1 [file ECE3-8-5164-s001.docx]

**Appendix 1. Model selection and output for sexually dimorphic species subset**

Table A1.1 Selection from a candidate list of Generalised Linear Mixed-effects Models for the likelihood of a bird reported to have died when recaptured in mist-nets in Britain & Ireland from 2005 to 2013 (for nine sexually dimorphic passerine species with at least 20 reported mortalities). All combinations of fixed effects listed and all models also included year, location (10km square) and family as categorical random effects. Three age classes were included (juvenile, post-juvenile first-calendar-year and after first-calendar-year). Time refers to continuous hour of day and Capture history to the number of previous captures for an individual. Sparrowhawk occurrence was a continuous variable describing the proportion of tetrads from the 10km square in which the capture site occurred where Sparrowhawk were recorded during the BTO Bird Atlas 2007-2011.

| **Response** | **Candidate models** | **Δ AICc** | ***w_i_*** | **df** |
| --- | --- | --- | --- | --- |
| Predated | *Age class + Month* | 0.00 | 1 | 16 |
|  | *Age class + Time* | 15.50 | 0 | 6 |
|  | *Age class* | 19.48 | 0 | 5 |
|  | *Age class + Capture history* | 21.29 | 0 | 6 |
|  | *Age class + Sex* | 21.38 | 0 | 6 |
|  | *Species + Age class* | 23.73 | 0 | 13 |
|  | *Species + Age class + Sex* | 25.72 | 0 | 14 |
|  | *Species + Time* | 94.49 | 0 | 12 |
|  | *Time + Sparrowhawk occurrence* | 96.06 | 0 | 5 |
|  | *Time* | 96.16 | 0 | 4 |
|  | *Species + Sparrowhawk occurrence* | 96.90 | 0 | 12 |
|  | *Species* | 97.11 | 0 | 11 |
|  | *Species + Capture history* | 97.45 | 0 | 12 |
|  | *Sex + Time* | 98.06 | 0 | 5 |
|  | *Sparrowhawk occurrence* | 98.58 | 0 | 4 |
|  | *Capture history* | 98.74 | 0 | 4 |
|  | *Species + Sex* | 99.06 | 0 | 12 |
|  | *Mass* | 100.15 | 0 | 4 |
|  | *Sex* | 100.59 | 0 | 4 |
|  | *Sex + Capture history* | 100.66 | 0 | 5 |
|  | *Species + Month* | 101.51 | 0 | 22 |
|  | *Time + Month* | 102.25 | 0 | 15 |
|  | *Time + Capture history + Month* | 102.63 | 0 | 16 |
|  | *Month + Sparrowhawk occurrence* | 103.97 | 0 | 15 |
|  | *Month* | 104.01 | 0 | 14 |
|  | *Sex + Month* | 106.18 | 0 | 15 |
|  |  |  |  |  |
| ‘Other’ | *Species + Age class + Sex* | 0.00 | 0.59 | 15 |
|  | *Species + Age class* | 0.73 | 0.41 | 14 |
|  | *Age class + Month* | 49.90 | 0 | 17 |
|  | *Age class + Capture history* | 51.96 | 0 | 7 |
|  | *Age class + Sex* | 52.60 | 0 | 7 |
|  | *Age class* | 53.33 | 0 | 6 |
|  | *Age class +Time* | 54.07 | 0 | 7 |
|  | *Species + Sex* | 66.65 | 0 | 13 |
|  | *Species + Time* | 67.55 | 0 | 13 |
|  | *Species* | 67.73 | 0 | 12 |
|  | *Species + Capture history* | 69.26 | 0 | 13 |
|  | *Species + Month* | 71.74 | 0 | 23 |
|  | *Sex + Month* | 121.97 | 0 | 16 |
|  | *Sex* | 122.52 | 0 | 5 |
|  | *Month* | 122.76 | 0 | 15 |
|  | *Sex + Time* | 123.76 | 0 | 6 |
|  | *Sex + Capture history* | 123.80 | 0 | 6 |
|  | *Time + Month* | 124.08 | 0 | 16 |
|  | *Time* | 124.96 | 0 | 5 |
|  | *Capture history* | 125.10 | 0 | 5 |
|  | *Time + Capture history + Month* | 125.21 | 0 | 17 |
|  | *Mass* | 125.47 | 0 | 5 |

Table A1.2 Output of Generalised Linear Mixed-effects Model for birds reported to have been predated from mist-nets (binary response) as a function of age class and month for a subset of passerine species which have sexually dimorphic plumage. There are three age classes, including, juveniles, first-year birds (excluding juveniles) and adults (all bird in second calendar year or older). Single model selected on basis of low AICc. Data from recaptured ringed birds reported to The British Trust for Ornithology from 2005 to 2013 in Britain & Ireland.

| **Model term** | **Estimate** | **SE** | **z** | **P** |
| --- | --- | --- | --- | --- |
| Intercept | -7.938 | 0.198 | -40.140 | <0.001 |
| Age_juv | 2.458 | 0.153 | 16.120 | <0.001 |
| Age_1y | 0.087 | 0.134 | 0.650 | 0.516 |
| Month_2 | -0.157 | 0.161 | -0.980 | 0.328 |
| Month_3 | -0.203 | 0.164 | -1.240 | 0.216 |
| Month_4 | -0.425 | 0.188 | -2.270 | 0.023 |
| Month_5 | -0.684 | 0.200 | -3.420 | 0.001 |
| Month_6 | -2.163 | 0.273 | -7.920 | <0.001 |
| Month_7 | -2.038 | 0.233 | -8.740 | <0.001 |
| Month_8 | -2.136 | 0.233 | -9.180 | <0.001 |
| Month_9 | -1.008 | 0.207 | -4.880 | <0.001 |
| Month_10 | -0.237 | 0.186 | -1.270 | 0.203 |
| Month_11 | 0.034 | 0.174 | 0.190 | 0.846 |
| Month_12 | -0.070 | 0.174 | -0.400 | 0.686 |

Table A1.3 Output of Generalised Linear Mixed-effects Model for birds reported to have died (binary response) during the capture and ringing process but not predated from mist-nets as a function of species and age class. There are three age classes, including, juveniles, first-year birds (excluding juveniles) and adults (all bird in second calendar year or older). Single model selected on basis of low AICc. Data from recaptured ringed birds reported to The British Trust for Ornithology from 2005 to 2013 in Britain & Ireland.

| **Model term** | **Estimate** | **SE** | **z** | **P** |
| --- | --- | --- | --- | --- |
| Intercept | -9.053 | 0.302 | 29.996 | <0.001 |
| Sp_Blackcap | -0.185 | 0.447 | 0.414 | 0.679 |
| Sp_Bullfinch | 1.885 | 0.282 | 6.674 | <0.001 |
| Sp_Chaffinch | 0.638 | 0.290 | 2.202 | 0.028 |
| Sp_Goldcrest | 1.582 | 0.317 | 4.993 | <0.001 |
| Sp_Goldfinch | 0.400 | 0.404 | 0.990 | 0.322 |
| Sp_Great_Tit | -0.180 | 0.277 | 0.649 | 0.516 |
| Sp_Greenfinch | 0.628 | 0.303 | 2.075 | 0.038 |
| Sp_Siskin | -0.150 | 0.400 | 0.376 | 0.707 |
| Age_juv | 1.880 | 0.194 | 9.676 | <0.001 |
| Age_1y | 0.360 | 0.185 | 1.943 | 0.052 |
| Sex_male | -0.126 | 0.150 | 0.842 | 0.390 |

**Appendix 2. Model selection and output for main analysis**

Table A2.1. Selection from a candidate list of Generalised Linear Mixed-effects Models for the likelihood of a bird reported to have died when recaptured in mist-nets in Britain & Ireland from 2005 to 2013 (for passerine species with at least 20 reported mortalities). All combinations of fixed effects listed and all models also included year, location (10km square) and family as categorical random effects. Three age classes were included (juvenile, post-juvenile first-year and after first-year). Time refers to continuous hour of day and Capture history to the number of previous captures for an individual. Sparrowhawk occurrence was a continuous variable describing the proportion of tetrads from the 10km square in which the capture site occurred where Sparrowhawk were recorded during the BTO Bird Atlas 2007-2011.

| **Response** | **Candidate models** | **Δ AICc** | ***w_i_*** | **df** |
| --- | --- | --- | --- | --- |
| Predated | *Age class + Month* | 0.00 | 1 | 17 |
|  | *Age class + Time* | 108.08 | 0 | 7 |
|  | *Species + Age class* | 124.84 | 0 | 24 |
|  | *Age class* | 133.02 | 0 | 6 |
|  | *Age class + Capture history* | 134.94 | 0 | 7 |
|  | *Time + Capture history + Month* | 219.16 | 0 | 17 |
|  | *Species + Time* | 219.66 | 0 | 23 |
|  | *Time + Month* | 221.77 | 0 | 16 |
|  | *Species + Month* | 226.99 | 0 | 33 |
|  | *Time* | 227.27 | 0 | 5 |
|  | *Time + Sparrowhawk occurrence* | 228.93 | 0 | 6 |
|  | *Month* | 237.93 | 0 | 15 |
|  | *Species + Capture history* | 238.37 | 0 | 23 |
|  | *Month + Sparrowhawk occurrence* | 239.61 | 0 | 16 |
|  | *Species* | 240.38 | 0 | 22 |
|  | *Species + Sparrowhawk occurrence* | 241.97 | 0 | 23 |
|  | *Capture history* | 244.18 | 0 | 5 |
|  | *Mass* | 247.30 | 0 | 5 |
|  | *Sparrowhawk occurrence* | 248.66 | 0 | 5 |
|  |  |  |  |  |
| ‘Other’ | *Species + Age class* | 0.00 | 1 | 24 |
|  | *Age class + Month* | 185.69 | 0 | 17 |
|  | *Species + Month* | 219.99 | 0 | 33 |
|  | *Species* | 223.34 | 0 | 22 |
|  | *Species + Time* | 224.10 | 0 | 23 |
|  | *Species + Capture history* | 225.30 | 0 | 23 |
|  | *Age class + Capture history* | 271.06 | 0 | 7 |
|  | *Age class* | 274.97 | 0 | 6 |
|  | *Age class + Time* | 276.96 | 0 | 7 |
|  | *Mass* | 447.90 | 0 | 5 |
|  | *Month* | 454.29 | 0 | 15 |
|  | *Time + Month* | 455.06 | 0 | 16 |
|  | *Time + Capture history + Month* | 457.03 | 0 | 17 |
|  | *Time* | 464.30 | 0 | 5 |
|  | *Capture history* | 464.95 | 0 | 5 |

Table A2.2. Output of Generalised Linear Mixed-effects Model for birds reported to have been predated from mist-nets (binary response) as a function of age class and month. There are three age classes, including, juveniles, first-year birds (excluding juveniles) and adults (all bird in second calendar year or older). Single model selected on basis of low AICc. Data from recaptured ringed birds reported to The British Trust for Ornithology from 2005 to 2013 in Britain & Ireland.

| **Model term** | **Estimate** | **SE** | **z** | **P** |
| --- | --- | --- | --- | --- |
| Intercept | -7.938 | 0.198 | -40.140 | <0.001 |
| Age_juv | 2.458 | 0.153 | 16.120 | <0.001 |
| Age_1y | 0.087 | 0.134 | 0.650 | 0.516 |
| Month_2 | -0.157 | 0.161 | -0.980 | 0.328 |
| Month_3 | -0.203 | 0.164 | -1.240 | 0.216 |
| Month_4 | -0.425 | 0.188 | -2.270 | 0.023 |
| Month_5 | -0.684 | 0.200 | -3.420 | 0.001 |
| Month_6 | -2.163 | 0.273 | -7.920 | <0.001 |
| Month_7 | -2.038 | 0.233 | -8.740 | <0.001 |
| Month_8 | -2.136 | 0.233 | -9.180 | <0.001 |
| Month_9 | -1.008 | 0.207 | -4.880 | <0.001 |
| Month_10 | -0.237 | 0.186 | -1.270 | 0.203 |
| Month_11 | 0.034 | 0.174 | 0.190 | 0.846 |
| Month_12 | -0.070 | 0.174 | -0.400 | 0.686 |

Table A2.3. Output of Generalised Linear Mixed-effects Model for birds reported to have died (binary response) during the capture and ringing process but not predated from mist-nets as a function of species and age class. There are three age classes, including, juveniles, first-year birds (excluding juveniles) and adults (all bird in second calendar year or older). Single model selected on basis of low AICc. Data from recaptured ringed birds reported to The British Trust for Ornithology from 2005 to 2013 in Britain & Ireland.

| **Model term** | **Estimate** | **SE** | **z** | **P** |
| --- | --- | --- | --- | --- |
| Intercept | -8.921 | 0.287 | -31.129 | <0.001 |
| Sp_Blackcap | -0.132 | 0.418 | -0.314 | 0.753 |
| Sp_Blue_Tit | 0.957 | 0.272 | 3.523 | <0.001 |
| Sp_Bullfinch | 1.727 | 0.306 | 5.644 | <0.001 |
| Sp_Chaffinch | 0.561 | 0.323 | 1.736 | 0.083 |
| Sp_Chiffchaff | 1.810 | 0.295 | 6.129 | <0.001 |
| Sp_Coal_Tit | 1.421 | 0.299 | 4.760 | <0.001 |
| Sp_Dunnock | -0.087 | 0.335 | -0.258 | 0.796 |
| Sp_Goldcrest | 1.642 | 0.345 | 4.752 | <0.001 |
| Sp_Goldfinch | 0.345 | 0.379 | 0.909 | 0.363 |
| Sp_Great_Tit | -0.410 | 0.303 | -1.352 | 0.176 |
| Sp_Greenfinch | 0.830 | 0.328 | 2.528 | 0.011 |
| Sp_Lesser_Redpoll | 1.583 | 0.367 | 4.309 | <0.001 |
| Sp_Long_tailed_Tit | 0.252 | 0.324 | 0.778 | 0.436 |
| Sp_Reed_Warbler | 0.328 | 0.340 | 0.967 | 0.334 |
| Sp_Robin | 0.089 | 0.321 | 0.277 | 0.781 |
| Sp_Siskin | -0.398 | 0.430 | -0.926 | 0.354 |
| Sp_Willow_Warbler | 1.775 | 0.324 | 5.473 | <0.001 |
| Sp_Wren | 1.193 | 0.303 | 3.934 | <0.001 |
| Age_juv | 1.431 | 0.088 | 16.209 | <0.001 |
| Age_1y | 0.328 | 0.097 | 3.379 | 0.001 |

**Appendix 3.**

Table A3. Identity of species reported to have attacked or predated birds recaptured in mist-nets from 2005 to 2013 in Britain & Ireland. Most common prey species only reported if a single species was ranked highest for a predator.

| **Identity** | **Scientific name** | **Frequency**  **reported** | **Most common prey (% of attacker total)** |
| --- | --- | --- | --- |
| Sparrowhawk | *Accipiter nisus* | 285 | Blue Tit (20%) |
| Unknown | -- | 95 | Great Tit (18%) |
| Kestrel | *Falco tinnunculus* | 83 | Blue Tit (20%) |
| Stoat | *Mustela erminea* | 39 | Greenfinch (18%) |
| Raptor sp. | *--* | 39 | Blue Tit (28%) |
| Domestic cat | *Felis catus* | 37 | Blue Tit (24%) |
| Magpie | *Pica pica* | 22 | Reed Warbler (14%) |
| Great Tit | *Parus major* | 16 | Blue Tit (50%) |
| Unknown mammal | -- | 13 | Robin (31%) |
| Great Spotted Woodpecker | *Dendrocopos major* | 11 | Blue Tit (45%) |
| Unknown bird | -- | 9 | Great Tit (33%) |
| Blue Tit | *Cyanistes caeruleus* | 8 | Blue Tit (25%) |
| Brown Rat | *Rattus norvegicus* | 8 | -- |
| Hymenoptera | -- | 6 | Long-tailed Tit (33%) |
| Carrion Crow | *Corvus corone* | 5 | -- |
| Weasel | *Mustela nivalis* | 5 | Great Tit (40%) |
| Red Fox | *Vulpes vulpes* | 4 | Fieldfare (50%) |
| Tawny Owl | *Strix aluco* | 4 | Robin (75%) |
| Common Buzzard | *Buteo buteo* | 3 | -- |
| Jackdaw | *Corvus monedula* | 3 | -- |
| Jay | *Garrulus glandarius* | 3 | Coal Tit (66%) |
| Little Owl | *Athene noctua* | 3 | Robin (66%) |
| Mallard | *Anas platyrhynchos* | 2 | Lesser Redpoll (100%) |
| Great Grey Shrike | *Lanius excubitor* | 2 | -- |
| Grey Squirrel | *Sciurus carolinensis* | 2 | Blue Tit (100%) |
| Hobby | *Falco subbuteo* | 2 | Sand Martin (100%) |
| Ferret | *Mustela putorius furo* | 2 | -- |
| Water Rail | *Rallus aquaticus* | 2 | -- |
| Bittern | *Botaurus stellaris* | 1 | Bearded Tit (100%) |
| Grey Heron | *Ardea cinerea* | 1 | Reed Warbler (100%) |
| Moorhen | *Gallinula chloropus* | 1 | Wren (100%) |
| Unknown corvid | -- | 1 | Great Tit (100%) |
